# The Binding of Human IgG to Minipig FcγRs – Implications for Preclinical Assessment of Therapeutic Antibodies

**DOI:** 10.1007/s11095-019-2574-y

**Published:** 2019-02-05

**Authors:** Jerome Egli, Tilman Schlothauer, Christian Spick, Stefan Seeber, Thomas Singer, Alex Odermatt, Antonio Iglesias

**Affiliations:** 1 Pharma Research and Early Development (pRED), Pharmaceutical Sciences, Roche Innovation Center Basel, Basel, Switzerland; 2 Pharma Research and Early Development (pRED), Pharmaceutical Sciences, Roche Innovation Center Munich, Munich, Germany; 3 Division of Molecular and Systems Toxicology, Department of Pharmaceutical Sciences, University of Basel, Basel, Switzerland

**Keywords:** antibody effector function, FcγR, Göttingen minipig, IgG, interaction map

## Abstract

**Purpose:**

The Göttingen minipig is a relevant non-rodent species for regulatory toxicological studies. Yet, its use with therapeutic antibodies has been limited by the unknown binding properties of human immunoglobulins (huIgG) to porcine Fc *gamma* receptors (poFcγR) influencing safety and efficacy readouts. Therefore, knowing IgG-FcγR interactions in the animal model is a prerequisite for the use of minipigs in preclinical safety and efficacy studies with therapeutic antibodies.

**Methods:**

Here, we describe the cloning and expression of poFcγRs and their interactions with free and complexed human therapeutic IgG1 by surface plasmon resonance and flow cytometry.

**Results:**

We show here that poFcγRIa, poFcγRIIa, and poFcγRIIb bind huIgG1 antibodies with comparable affinities as corresponding huFcγRs. Importantly, poFcγRs bind huIgG immune complexes with high avidity, thus probably allowing human-like effector functions. However, poFcγRIIIa binds poIgG1a but not to huIgG1.

**Conclusions:**

The lack of binding of poFcγRIIIa to huIgG1 might cause underestimation of FcγRIIIa-mediated efficacy or toxicity as mediated by porcine natural killer cells. Therefore, the suitability of minipigs in preclinical studies with human therapeutic antibodies has to be assessed case by case. Our results facilitate the use of Göttingen minipigs for assessment of human therapeutic antibodies in preclinical studies.

**Electronic supplementary material:**

The online version of this article (10.1007/s11095-019-2574-y) contains supplementary material, which is available to authorized users.

## Introduction

Fc *gamma* Receptors (FcγRs) are a family of gylcoproteins expressed on the surface of leukocytes. They interact with the fragment crystallizable (Fc) part of immunoglobulin G (IgG) antibodies and trigger a variety of effector functions including antibody-dependent cellular cytotoxicity (ADCC), antibody-dependent cellular phagocytosis (ADCP), antigen internalization and presentation, or inflammatory cytokine release (1). The set of FcγRs of most mammalian species consists of the high affinity FcγRIa (CD64), low affinity FcγRIIa (CD32a) and FcγRIIIa (CD16), and the inhibitory FcγRIIb (CD32b) (2). Their cellular distribution and distinct affinities towards different IgG subclasses influence immune cell activation and control their effector functions upon IgG binding. Many novel therapeutic antibodies are IgG Fc engineered to alter the FcγR binding in order to achieve enhanced activity via ADCC or ADCP or to reduce effector function-mediated toxicity (3,4). Often, antibody effector functions are mediated upon interactions of low affinity FcγRs with immune complexes (IC). For example, IC formed by bevacizumab binding to vascular endothelial growth factor (VEGF) can lead to FcγRIIa-mediated platelet activation (5) and thrombosis in FcγRIIa transgenic mice (6). Thus, it is very important to characterize the binding of free- and immune-complexed IgG to different FcγRs as this can dramatically influence safety and efficacy.

The porcine species (*Sus scrofa*) is an increasingly used animal model for biomedical research. In particular the Göttingen minipig has gained importance for preclinical safety and efficacy studies due to its high similarity to the human (7,8). Also, the regulatory acceptance of the minipig as a relevant animal model for toxicological studies with biotherapeutics is growing (9). Furthermore, handling, housekeeping, and breeding of minipigs are much easier and cheaper than of non-human primates (NHP). So far, the Göttingen minipig has already been used for immunogenicity studies with infliximab and adalimumab (10). Presently, only few other minipig studies are performed with therapeutic antibodies (11) due to lacking knowledge about their pharmacology (12). Therefore, the importance of an adequate immunological characterization of the Göttingen minipig as a non-rodent species is widely recognized and promoted (13). The evaluation of the interactions of human therapeutic antibodies with porcine FcγRs (poFcγRs) is a basic requirement for the use of the minipig in preclinical studies. So far, only functional binding studies of poFcγRIa and variants of poFcγRIIb to porcine total IgG have been reported confirming the conserved function of these receptors in pigs (14,15). We have recently annotated the complete low affinity *FCGR* locus of the minipig including the localization of all poFcγR genes and the description of the hitherto unknown poFcγRIIa (16). Binding and function of NHP or mouse FcγRs interacting with human IgG (huIgG) were studied to assess cross-reactivity and to estimate the translation potential of this preclinical species (17–19). To our knowledge, no extensive studies investigating the interactions of huIgG to poFcγRs were performed for any porcine species. Thus, the lacking knowledge of the binding properties of huIgG to poFcγRs is still limiting the use of the minipig as a preclinical species with human therapeutic antibodies.

In the present work we hypothesized minipigs as a useful alternative for preclinical studies with therapeutic antibodies. Therefore, we qualitatively characterize the binding of human therapeutic antibodies to all FcγRs in the minipig. Furthermore, we assessed the binding of free- and immune-complexed huIgG1 antibodies to poFcγRs in comparison to huFcγRs. The data provide first insights into possible effector functionalities of human immunoglobulins in preclinical studies in minipigs.

## Materials and Methods

### Recombinant FcγRs and Antibodies

#### Cloning

Soluble FcγRs were designed as dimeric IgG Fc fusion proteins. Extracellular domains of poFcγRIa (UniProtKB: Q461Q0), poFcγRIIa (XM_021089520.1; 205Y), poFcγRIIb (UniProtKB: Q461P7), poFcγRIIb1 (UniProtKB: B9VVN4), poFcγRIIIa (UniProtKB: Q28942) as well as huFcγRIa (UniProtKB: P12314), huFcγRIIa (UniProtKB: P12318 R131), huFcγRIIb (UniProtKB: P31994), huFcγRIIIa (UniProtKB: P08637 V158) were used. The sequences were back translated, codon optimized, and ordered as gene syntheses from GeneArt (Invitrogen). Subsequently they were cloned into an expression vector containing the signal peptide from mouse Ig heavy chain variable region, an Avi biotinylation tag (GLNDIFEAQKIEWHE, Avidity), a His6 tag, and an IgA protease cleavage site (VVAPP’AP). The vector also contained inert huIgG1 (PGLALA) Fc parts allowing the dimerization of the FcγR extracellular domains by the expression as Fc fusion proteins (20). These constructs are referred to as soluble FcγRs hereafter.

Full-length poFcγRIa (amino acids (aa) 16-346), poFcγRIIa (205Y; aa 46-274), poFcγRIIb (aa 46-297), poFcγRIIb1 (aa 46-316), and poFcγRIIIa (aa 20-257) contained the human CD33 signal peptide (MPLLLLLPLLWAGALA) and a FLAG-tag (DYKDDDDK) at the N-terminus. Full-length huFcγRs, the human Fc receptor common *gamma* chain (FcR-γ chain), and the poFcR-γ chain (UniProtKB: Q9XSZ6) were designed without the FLAG-tag.

PoIgG1a (GenBank: U03781.1) and poIgG3 (GenBank: EU372658.1) heavy chain and Ig-*kappa* light chain (21) constant regions were coupled to the variable regions of the anti-human epidermal growth factor receptor 2 (HER2) antibody trastuzumab heavy chain (DrugBank: DB00072; aa 1-120) and Ig-*kappa* light chain (DrugBank: DB00072; aa 1-108), respectively. The correct transitions between the variable and the constant region of both antibodies were confirmed by molecular modeling. The recombinant antibodies contained the mouse Ig heavy chain V region 3 signal peptide (MGWSCIILFLVATATGVHS) and a C-terminal Avi biotinylation tag (GLNDIFEAQKIEWHE, Avidity). The resulting HER2 specific poIgG constructs are named poIgG1a-HER2 and poIgG3-HER2 hereafter.

The sequences of all constructs were verified prior to expression by DNA-sequencing (SequiServe and Microsynth).

### Expression

Soluble FcγRs and poIgGs were expressed in human embryonic kidney 293F (HEK293F) suspension cells cultured in shaker flasks (120 rpm, 37°C, 5% CO_2_, 85% humidity) using F17 expression medium supplemented with Pluronic and GlutaMAX (Gibco). Plasmids coding for FcγRs were transfected alone and poIgG heavy chains were co-transfected in equimolar ratio with plasmids coding for poIg-*kappa* light chain. Transient transfection was performed using 293free (Merck Millipore) premixed with OptiMEM (Gibco) and expression was enhanced by feeding and addition of valproic acid. The fed-batch culture was harvested by centrifugation 7 days after transfection and the supernatant was cleared by filtration.

Full length FcγRs were transiently expressed using the Expi293 system (Thermofisher). Suspension cells were seeded in 6 well-plates (120 rpm, 37°C, 5% CO_2_, 85% humidity) and co-transfected with porcine or human FcγRs together with the related FcR-γ chain in an equimolar ratio. The transfected cells were used 48 h post transfection.

### Purification and Analysis

Soluble FcγRs and poIgGs were purified by protein A (MabSelect SuRe, GE Healthcare) or, in the case of soluble FcγRIa, by nickel (HisTrap HP, GE Healthcare) affinity chromatography using the ÄKTAexplorer 100 Air system (GE Healthcare). Soluble FcγRs were further purified by preparative size exclusion chromatography (SEC) using a HiLoad 26/600 Superdex prep grade column (GE Healthcare) with 20 mM MOPS, 150 mM NaCl, pH 6.0 as a running buffer.

Purified proteins were quantified on a Nanodrop spectrophotometer (Thermo Scientific) and analyzed under reducing and non-reducing conditions by capillary gel electrophoresis using Caliper LabChip (Perkin Elmer) or sodium dodecyl sulfate polyacrylamide gel electrophoresis (SDS-PAGE) with NuPAGE 4–12% Bis-Tris gels in MES buffer followed by Coomassie staining (SimplyBlue, ThermoFisher). Aggregation and molecular weight of the FcγR products were determined by SEC coupled to Multi-Angle Light Scattering (MALS) using a Superdex 200 increase 10/300 GL column (GE Healthcare).

### Biotinylation

Soluble poFcγRs were biotinylated via the Avi-tag using the BirA Biotin-Protein Ligase standard reaction kit (Avidity). The biotinylation efficacy was assessed by liquid chromatography – mass spectrometry (LC-MS) after deglycosylation with PNGase F.

### Generation of FcγRIIa and FcγRIIa/b Specific Antibodies

Purified soluble poFcγRIIa, poFcγRIIb, and poFcγRIII were sent to BioRad for the generation of bivalent Fab antibodies dimerized via alkaline phosphatase containing FLAG and His6 epitope tags (Fab-A-FH). Binders were selected via phage display method (CysDisplay®) on BioRads Human Combinatorial Antibody Libraries (HuCAL). PoFcγRIIa/FcγRIIb cross-reactive HuCAL antibodies were generated by using poFcγRIIa as an antigen and poFcγRIII as a closely related antigen to prevent further cross reactivity. Similarly, poFcγRIIa specific antibodies were generated by using poFcγRIIb as a closely related antigen. All binders (HuCAL clones) were tested for their specificity by enzyme-linked immunosorbent assay (ELISA) coated with porcine FcγRIIa, FcγRIIb, and FcγRIII.

### Immune Complex Generation

IC were generated by overnight incubation at room temperature of the huIgG1 therapeutic antibody bevacizumab (149 kDa; Roche) and its dimerized target VEGF165 (38 kDa, BioLegend), as described earlier (5). The antibody to target ratio of 1:2.5 was generated using 4 μM bevacizumab and 10 μM dimerized VEGF165, whereas the ratio of 1:0.5 was generated using 20 μM bevacizumab and 10 μM VEGF165, and the ratio of 1:0.1 using 20 μM bevacizumab and 2 μM VEGF165. IC formation was analyzed by SEC-MALS using a HPLC system equipped with a Superdex 200 increase 10/300 GL column (GE Healthcare), a TREOS laser light scattering detector, and a T-rEX differential refractometer (Wyatt Technology).

### Flow Cytometry

#### Phenotyping of FcγR Expression

FcγR expression was assessed in whole blood of a Göttingen minipig sampled in K2EDTA Vacutainer tubes (BD) followed by treatment with lysing buffer (BD PharmLyse) to remove erythrocytes. Minipig blood cells were stained with PE-conjugated antibodies against porcine CD16 (clone G7, BioRad) or unconjugated HuCAL antibodies against poFcγRIIa (clone AbD29332.1 “HuCAL32”), FcγRIIa/b (clone AbD32591.1 “HuCAL91”), or the isotype control Fab-A-FH (clone AbD05930). The FcγR expression of transfected HEK293F cells was assessed by staining using the abovementioned antibodies or the PE-conjugated antibodies against human CD64 (clone 10.1, BioLegend), human CD32 (clone 3D3, BD), human CD16 (clone 3G8, BD), or FLAG tag (clone L5, BioLegend). Unconjugated antibodies were detected using the secondary PE conjugated goat F(ab’)2 anti-huIgG after washing with FACS buffer (Dulbecco’s phosphate-buffered saline (DPBS, Gibco) containing 2% bovine serum albumin (Sigma) and 0.1% sodium azide (Sigma)). After washing with DPBS, dead cells were stained by the amine reactive Zombie Aqua dye (BioLegend) and the preparations were fixed using BD CellFIX. Events were acquired on BD LSRFortessa with BD FACSDiva software and data was further analyzed using FlowJo.

#### Immune Complex Binding

Binding of IC was assessed by flow cytometry analysis on whole blood of three Göttingen minipigs. Fresh blood was collected in Vacutainer tubes coated with K2EDTA (BD) and subsequently treated with erythrocyte lysis buffer (PharmLyse, BD) and washed with DPBS. The remaining blood cells were incubated with the amine reactive dye Zombie Aqua (BioLegend). After washing with FACS buffer, the blood cells were incubated in 96 well plates for 1 h at 4°C with different concentrations of bevacizumab, or bevacizumab-VEGF165 IC diluted in FACS buffer. Bevacizumab to VEGF165 ratios of 1:2.5, 1:0.5, and 1:0.1 were used. Unbound antibodies or complexes were removed by intensive washing with FACS buffer. PE-conjugated secondary goat F(ab’)2 antibodies against huIg-*kappa* (Biorad) were used to detect membrane-bound bevacizumab or IC. After another two washes with FACS buffer, 100′000 events were recorded on BD LSRFortessa with and the software BD FACSDiva. Data was further analyzed using FlowJo.

### SPR Experiments

#### IgG Capturing Setup

The interaction of porcine or human FcγR variants to porcine or human IgG anti-HER2 was analyzed using surface plasmon resonance (SPR) on a Biacore T200 system (GE Healthcare). First, the extracellular domain of HER2 was immobilized at pH 4.5 to >3000 response units (RU) on a CM5 chip using the amine coupling kit (GE Healthcare). Then, the HER2 specific antibodies trastuzumab (huIgG1, Roche), poIgG1a-HER2 and poIgG3-HER2 were injected at a concentration of 100 nM in PBS-P+ buffer (GE Healthcare) with a pulse of 30s at a flow rate of 10 μl/min reaching capturing levels of 1000RU. Soluble porcine or human FcγRs were prepared in solutions of 600, 200 nM and 66.7 nM in PBS-P+ and applied at a flow rate of 30 μl/min for 90s. The dissociation phase was monitored for 600 s followed by regeneration of the surface by a 60s and 20s washing step with a 10 mM Glycine pH 2.1 at a flow rate of 10 μl/min. All experiments were performed in PBS-P+ pH 7,4 running buffer.

#### FcγR Capturing Setup

An alternative setup was used to compare binding of poFcγRs to free- and immune-complexed huIgG1. Biotinylated soluble poFcγRs were reversibly captured on a CAP chip using the standard Biotin CAPture reagent kit (GE Healthcare) at pH 7.4 PBS-P+. The capturing level of FcγR variants reached 940–2543 RU. Porcine or human biotinylated FcγR variants were prepared as solution of 200 nM in PBS-P+ and captured with a pulse of 180 s at a flow rate of 5 μl/min. Subsequently, human free- or immune-complexed IgG1 were applied at a concentration of 600, 200 and 66.7 nM in PBS-P+ at a flow rate of 30 μl/min for 120 s. The dissociation phase was monitored for 600 s. Then, the surface was regenerated by a 120 s washing step with the regeneration solution for the CAP chip (GE Healthcare) at a flow rate of 10 μl/min.

#### SPR Data Analysis

The Biacore T200 software (GE Healthcare) was used to evaluate data from SPR experiments and to display binding curves. Interaction Map was used to separate heterogeneous binding into its individual 1:1 interactions with different kinetics. For this, data from SPR experiments were imported into TraceDrawer software (Ridgeview Instruments AB) and further processed with the Interaction Map program (Ridgeview Instruments AB).

## Results

Interactions between IgG antibodies and their Fc receptors are of high complexity. To obtain a thorough characterization, we studied poFcγRs as recombinant soluble proteins and expressed on the cell surface, as well as minipig blood cells that natively express FcγRs. Interactions of poFcγRs were assessed with different free- or immune-complexed IgG antibodies and therapeutics.

### Binding of huIgG to poFcγRs

The purpose of this experiment was to show qualitative binding of poFcγRs to huIgG1, the most commonly used therapeutic human antibody isotype, by SPR. A highly sensitive assay is needed to detect weak interactions because low affinity FcγRs (FcγRIIa, FcγRIIb, and FcγRIIIa) generally interact only weakly with free IgG. Therefore, soluble porcine and human FcγRs were designed and used here as dimers of FcγR extracellular domains expressed as inert Fc fusion proteins. The dimeric structure provides an avidity effect and increases the molecular mass leading to higher sensitivity and therefore allowing a qualitative binding analysis (20). Transient expression in HEK293F cells and subsequent purification yielded soluble FcγRs of >98% purity as determined by capillary gel electrophoresis or SDS-PAGE (not shown). PoFcγRIIb is exclusively composed of dimers, whereas poFcγRIa, poFcγRIIa, and poFcγRIIIa preparations additionally contained 32%, 74%, and 77% aggregates, respectively, even after SEC purification (not shown). N-linked glycosylation of FcγRs and Fc fusion was effective in HEK293F cells as observed by PNGase F digestion followed by SDS-PAGE (Fig. S1).

**Fig. 1:**
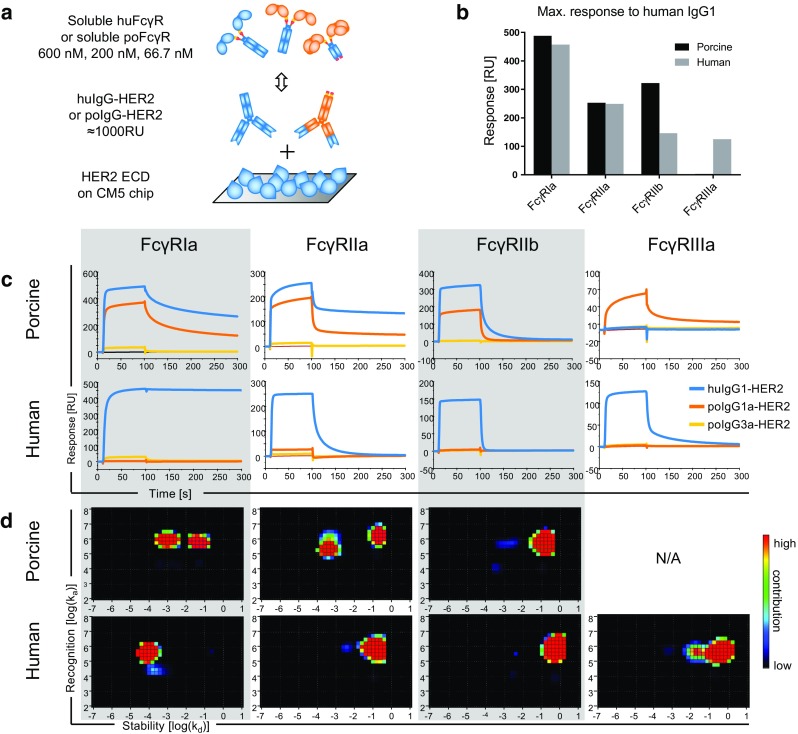
SPR binding analysis of soluble FcγRs to IgG. (**a**) Scheme depicting the assay setup. First, extracellular domains of HER2 were coated on a CM5 sensor chip. Then, HER2 specific human (blue structures) and porcine (orange structures) antibodies were captured on different flow cells. Their interactions with soluble porcine or human FcγRs were measured. The drawing shows low affinity FcγRs with two and the high affinity FcγRIa with three extracellular domains (oval shapes). (**b**) The graph shows the maximum response of 600 nM porcine (black bars) and human (grey bars) FcγRs obtained with huIgG1. (**c**) Real-time sensorgrams from SPR analysis. Interaction of IgG to poFcγRs is shown in the upper row and to huFcγRs in the lower row whereas the respective FcγRs are named above. Binding of 600 nM soluble FcγRs to trastuzumab (huIgG1, blue line), poIgG1a-HER2 (orange line), and poIgG3-HER2 (yellow line) is shown. Only the highest concentration of the titration with 600, 200, and 66.7 nM of soluble FcγRs is shown for clarity. (**d**) Interaction Map analysis resulting from trastuzumab binding to all concentrations of porcine and human FcγRs is shown in the upper and lower row, respectively. The binding is separated in several parallel interactions with unique kinetics, as displayed by spots on a graph with k_d_ on the x-axis and k_a_ on the y-axis. The heat map is a measure of the contribution from red = high to blue = low of each interaction to the total binding. No interaction was detected with poFcγRIIIa; therefore, it could not be analyzed (N/A).

For the SPR binding analysis, the recombinant HER2 antigen was coated on a CM5 sensor chip and then allowed to capture trastuzumab, a HER2 specific huIgG1 therapeutic antibody, or HER2 specific poIgGs (Fig. 1a). To this purpose, the two most abundant isotypes in porcine blood, IgG1a and IgG3 (22), were recombinantly expressed with HER2 specificity, and named poIgG1a-HER2 and poIgG3-HER2, respectively. The soluble poFcγRs were then allowed to bind human and porcine HER2-specific IgG (Fig. 1a).

Figure 1b shows the maximum responses observed with huIgG1 in interaction with 600 nM of porcine and human FcγRs. From this analysis we conclude that trastuzumab binds to most poFcγRs in a similar magnitude as to huFcγRs (Fig. 1b). A closer analysis of the sensorgrams generated using three concentrations of the soluble FcγRs permits a ranking of the binding strength among the different FcγRs (Fig. 1c). The sensorgrams show the response (RU) during association of the soluble FcγRs to antigen bound IgG until the steady state in the first 100 s followed by their dissociation. Among poFcγRs, we identified poFcγRIa as the strongest binder for huIgG1 based on the quicker association and the slower dissociation, followed by poFcγRIIa. PoFcγRIIb is the weakest binder with the quickest dissociation whereas poFcγRIIIa did not bind huIgG1. However, the functionality of poFcγRIIIa was demonstrated through its binding to poIgG1 (Fig. 1c). A similar binding pattern was observed for huFcγRs with huFcγRIa as the strongest binder of huIgG1 followed by huFcγRIIa, FcγRIIb, and huFcγRIIIa in a similar range. Comparing the orthologous porcine and human FcγRs, huIgG1 bound stronger to poFcγRIIa and poFcγRIIb but weaker to poFcγRIa and poFcγRIIIa as compared to the human orthologue (Fig. 1c). For all poFcγRs, except poFcγRIIIa, we observed a similar binding pattern of huIgG1 and poIgG1a-HER2. In contrast, poIgG3-HER2 showed only weak interactions to poFcγRIa and poFcγRIIa and no binding to poFcγRIIb and poFcγRIIIa. Vice versa, huFcγRs did not notably bind to poIgGs (Fig. 1c). In sum, we found that poIgG1 binds to poFcγRIa > poFcγRIIa > poFcγRIIb and poFcγRIIIa in a similar range, whereas huIgG1 binds to poFcγRIa > poFcγRIIa > poFcγRIIb > > poFcγRIIIa.

The shape of the sensorgrams in Fig. 1c suggested complex multiple interactions contributing to IgG-FcγR bindings. Such heterogeneous interactions probably originate from different qualities of the individual FcγRs based on their integrity and the presence of aggregates. To assess the contribution of quality issues leading to heterogeneous interactions, we also analyzed the huIgG1 binding data using the Interaction Map method (Fig. 1d). It allows the decomposition of time-resolved binding curves into separate interactions with unique combinations of association rates k_a_ [M^−1^, s^−1^] and dissociation rates k_d_ [s^−1^], contributing to the total binding (23). Therefore, the Interaction Map analysis allows addressing the heterogeneity of IgG-FcγR interactions. The resulting on-off plots display single interactions by their dissociation (log(k_d_), x-axis) and association (log(k_a_), y-axis) values colored according to their contribution to the total binding (Fig. 1d). Because no interaction of trastuzumab was observed with poFcγRIIIa, this data could not be analyzed by Interaction Map. For the other FcγRs, this analysis disclosed multiple interactions involved in the binding of huIgG1 to poFcγRIa, poFcγRIIa, and huFcγRIIIa (Fig. 1d). Interestingly, the FcγRs with the most obvious multivalent binding properties were the preparations with the highest proportion of aggregates. Therefore, one spot originates from the bivalent functional binding, whereas the other spot reflects the binding to aggregates contained in the preparation. Because aggregates reformed after SEC purification, it was not possible to identify which interaction was responsible for the functional binding. The correct binding kinetics of these FcγRs must be a mixture of the observed interactions. Therefore, we refrain from reporting affinities based on one 1:1 kinetic. Additionally, it was shown by other authors that IgG-FcγR interactions do not depend on only one 1:1 kinetic and are strongly influenced by the experimental setup and other factors, such as FcγR glycosylation (24).

In addition to poFcγRIIb, another isoform named poFcγRIIb1 has been reported having a 19 amino acid in-frame insertion in the cytoplasmic domain. Apart from the signal sequence, these variants also differ by one polymorphism in the extracellular domain 1 and two polymorphisms in the extracellular domain 2 (the latter are marked in yellow in Fig. S2) (25). We directly compared these two polymorphic variants in SPR regarding binding to porcine or human IgG and found no differences in IgG isotype selectivity and negligible stronger binding of the poFcγRIIb1 variant (Fig. S3).

### Binding of huIgG1 to poFcγRs on Cells

Next, we addressed binding of free huIgG1 to poFcγRs in a more biological system with transfected HEK293F cells expressing surface-anchored FcγRs. Due to the lack of available antibodies specific for poFcγRs, we also generated phage-display based recombinant antibodies with specificity for poFcγRs using the HuCAL technology. The specificity of these HuCAL antibodies was also assessed using cell surface-anchored FcγRs.

**Fig. 2:**
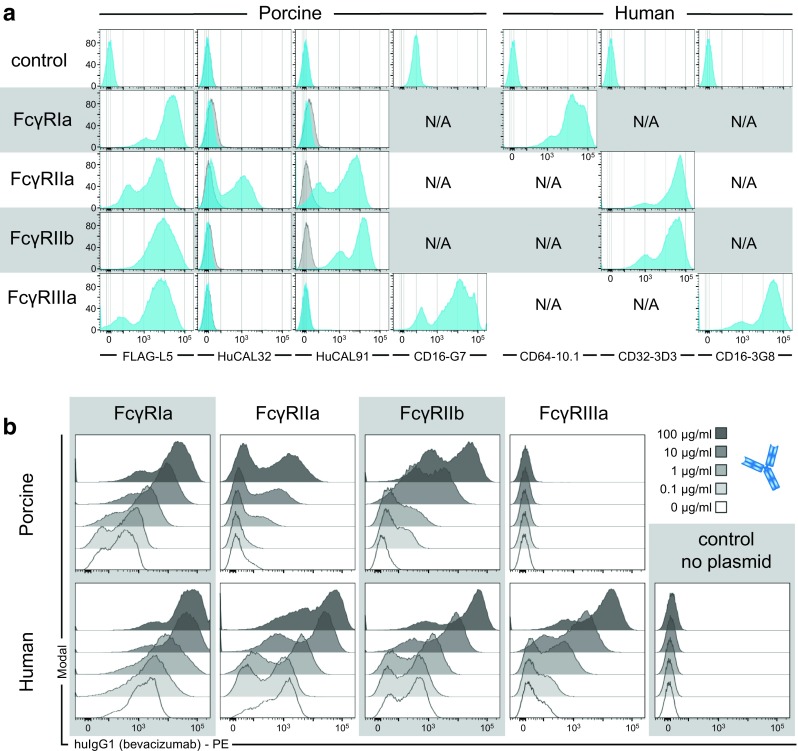
Binding of bevacizumab (huIgG1) to FcγRs transiently expressed on HEK293F cells. (**a**) HEK293F cells expressing the indicated porcine (left panel) and human (right panel) FcγRs were analyzed by flow cytometry using the antibodies indicated below each column. HEK293F cells transfected without plasmid served as a negative control (first row). Blue histograms show binding of the antibody to the respective cells whereas N/A indicates combinations of antibodies and FcγRs that were not analyzed. Overlaid grey histograms display the staining with the HuCAL control antibody. (**b**) Bevacizumab was titrated and incubated with HEK293F cells expressing the indicated FcγR. After intense washing, FcγR-bound IgG was stained with PE-conjugated goat F(ab’)2 anti-huIg-*kappa* secondary antibody and analyzed by flow cytometry. Stacked histograms show binding of increasing concentrations with increasing intensity: no bevacizumab (open histogram), 0.1 μg/ml (shaded in light grey), 1, 10, and 100 μg/ml (shaded in dark grey).

Full length poFcγRs with extracellular FLAG tags encoded at the N-terminus were transiently expressed on HEK293F cells. However, full-length huFcγRs were expressed without FLAG tags. The data shown in Fig. 2a demonstrate expression of all porcine and human FcγRs on the cell surface of HEK293F cells. The expression of huFcγRs and of poFcγRIIIa was characterized via commercial FcγR-specific antibodies whereas a FLAG tag specific antibody was used to characterize the expression of all poFcγRs. The expression of poFcγRIIa was further demonstrated with the antibody clone HuCAL32 that binds specifically to this FcγR in contrast to the antibody clone HuCAL91 that is cross-reactive to the closely similar FcγRIIb (Fig. 2a).

Binding to FcγRs expressed on HEK293F cells was then assessed using different concentrations of bevacizumab, a huIgG1 anti-VEGF therapeutic antibody displaying similar SPR binding to poFcγR as trastuzumab (not shown). Cell bound bevacizumab was detected via flow cytometry using goat F(ab’)2 anti-huIg-*kappa* secondary antibody. The results show a concentration-dependent binding of bevacizumab to porcine (except for poFcγRIIIa) and human FcγRs (Fig. 2b). From these data we conclude that surface-anchored poFcγRIa, IIa and IIb, but not poFcγRIIIa can bind by free huIgG1.

### Binding of huIgG1 Immune Complexes to poFcγRs

Human low affinity FcγRs mediate their functions rather via interaction with IC in contrast to free IgG (26). The increase in avidity compensates for the low affinity and allows stable binding to the IC ultimately leading to activation of the FcγRs. In order to assess binding of poFcγRs to huIgG1 IC we performed SPR experiments with pre-formed IC of bevacizumab and its dimeric target antigen VEGF.

**Fig. 3:**
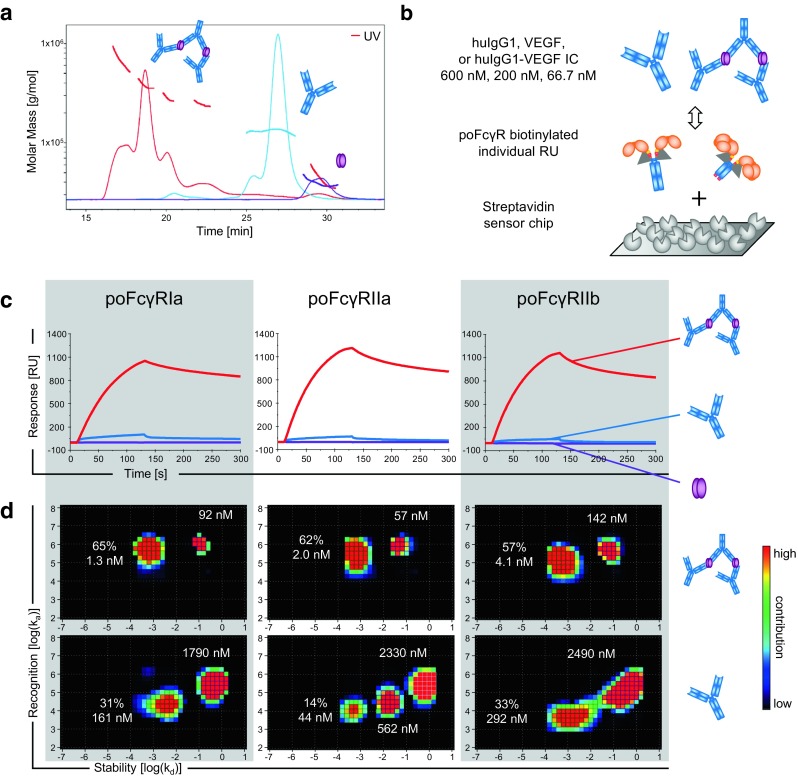
Comparison of free bevacizumab and IC by binding to poFcγRs using SPR. (**a**) SEC-MALS analysis shows the molecular weight of free bevacizumab (blue line), VEGF (purple line), and complexes formed by bevacizumab and VEGF in a molar ratio of 1:5 (red line). (**b**) Biotinylated (grey triangles) soluble poFcγRs were captured with different densities on a streptavidin senor chip. Interactions with free bevacizumab (blue) and IC with VEGF dimers (purple ovals) were measured by SPR. Three different concentrations of IC and free huIgG1 with the same amount of IgG were assayed (600, 200, 66.7 nM). PoFcγRIIIa was excluded from this experiment due to insufficient biotinylation. (**c**) SPR sensorgrams resulting from binding of the above indicated FcγR to the highest concentration of VEGF (purple line), bevacizumab (blue line), and IC (red line) are shown. (**d**) Binding curves from panel C were resolved by the Interaction Map method. Binding of huIgG1 IC to the FcγR indicated above each column is shown in the upper row and binding of free huIgG1 is shown below. The equilibrium binding constant K_D_ [nM] is indicated next to each major interaction spot and its contribution [%] to the total binding is indicated for the spot with the highest affinity.

To generate physiological IC, bevacizumab was co-incubated with VEGF and the resulting complexes were studied by SEC-MALS. The stoichiometric ratio of one antibody together with an excess of five VEGF dimers resulted in large IC without remaining free IgG where the majority of complexes is composed of three or more antibodies (Fig. 3a). For the measurement of their binding profiles in comparison to free IgG, all poFcγRs were biotinylated and coated on the sensor chip (Fig. 3b). For every FcγR, two different capturing densities were assayed. The densities of FcγRIa (940RU), FcγRIIa (1020RU), and FcγRIIb (2543RU) were found to give best results probably reflecting their different affinities to huIgG1. We, however, did not achieve sufficient biotinylation of poFcγRIIIa to increase its capturing density above 54RU, and was therefore excluded from the experiment. Subsequently, 600, 200, and 66.7 nM of free huIgG1 or IC formed with the same amount of huIgG1 were used to assess the binding strength (Fig. 3b).

The sensorgrams in Fig. 3c show a strong increase in the maximum response and a more stable interaction with IC compared to free huIgG1 in all poFcγRs. We again analyzed the SPR binding data with the Interaction Map method (Fig. 3d) first, because the observed maximum response largely depends on the size of the bound complex and second, because we expect avidity based heterogeneous IC-FcγR interactions. Using this setup, we observed two to three interactions contributing to the binding of poFcγRs to free huIgG1, probably resulting from partial activity of the soluble FcγRs (Fig. 3d). The contribution of all interactions shifted towards lower k_d_ and higher k_a_ values and ultimately towards a stronger binding comparing free bevacizumab to IC. Additionally, Fig. 3d shows a shift of the individual interactions towards a higher affinity. For poFcγRIa, for example, the higher affinity interaction shifts the center of the spot to a 5x longer half-life (shift towards lower k_d_) as seen in Fig. 3d and evaluated by the interaction map software, a 10x quicker association (shift towards higher k_a_), and therefore a 100x enhanced affinity (K_D_ = k_d_/k_a_) comparing free huIgG1 to IC.

Additionally, its contribution increases from 31% to 65%. On the other hand, the low affinity interaction decreases its contribution from 50% to 16% (Fig. 3d, left plots). For poFcγRIIa and poFcγRIIb the changes in affinity from free huIgG1 to IC are comparable to those for poFcγRIa. This data clearly demonstrates a stronger and more stable interaction of huIgG1 IC with poFcγRs than with free huIgG1 based on avidity effects. IC binding is a prerequisite for effector functions triggered by huIgG in minipigs.

### Binding of huIgG1 IC to Minipig Blood Cells

**Fig. 4:**
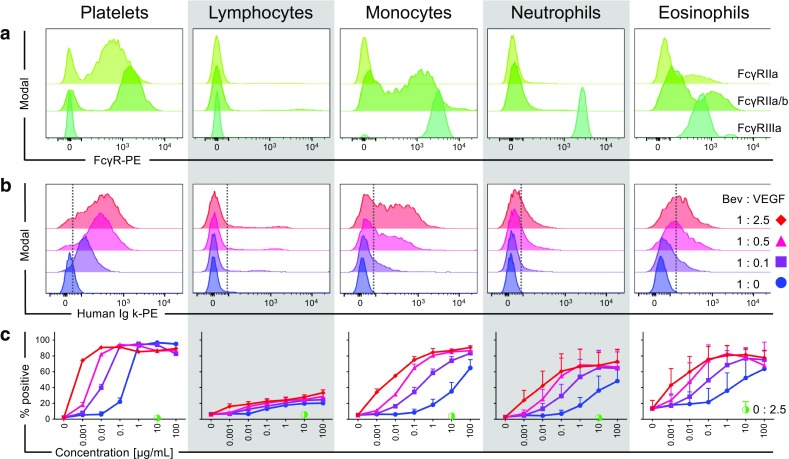
Binding of free huIgG1 (bevacizumab) and IC to minipig whole blood in comparison to the FcγR expression. FcγR expression and huIgG binding was assessed by flow cytometry in whole blood of Göttingen minipigs. The cell types were gated from single live cells by their FSC and SSC properties as described in detail in Fig. S4. (**a**) Histograms show the expression of poFcγRIIa (HuCAL32, light green histogram), poFcγRIIa/b (cross reactive HuCAL91, green histogram) and poFcγRIIIa (clone G7, dark green histogram) in platelets, lymphocytes, monocytes, neutrophils and eosinophils (from left to right). (**b**) Stacked histograms show the binding of 0.1 μg/ml free huIgG1 (blue) and the same amount of huIgG1 complexed using 0.1 parts of VEGF165 (purple), 0.5 parts of VEGF165 (magenta), or 2.5 parts of VEGF165 to the different minipig blood cell subsets. The dotted line represents the gate separating PE-negative (left) from PE-positive (right) events. (**c**) Graphs show the percentage of PE-positive cells with increasing concentrations of free- (blue circles) and immune-complexed bevacizumab with concentrations ranging from 100 to 0.001 μg/ml of huIgG1 and a control containing 0 μg/ml bevacizumab or IC. IC generated by the following antibody to target ratios are displayed: 1:0.1 (purple squares), 1:0.5 (magenta triangles), 1:2.5 (red diamonds), and VEGF alone (half-filled green circle). Error bars represent the standard deviation within one representative experiment using three minipigs. Multiple experiments with IC (ratio 1:2.5) using a total of seven minipigs led to similar results.

Next, we studied interactions of free huIgG1 (bevacizumab) to blood cells of Göttingen minipigs that natively express poFcγRs. Free huIgG1 and different preparations of IC were titrated and co-incubated with minipig whole blood for 1 h at 4°C in FACS buffer containing sodium azide to prevent internalization. Bound antibodies or IC were stained using goat F(ab’)2 anti-huIg-*kappa* secondary antibody and analyzed by flow cytometry. The different blood cell subsets were gated from forward and side scatter (FSC/SSC) of viable single cells without including specific cell surface markers due to limited availability of specific antibodies, fluorochromes, and cross-reactivity. The gating strategy and identity of the different cell types of the minipig blood is shown in Fig. S4. The FcγR expression in the respective minipig blood cells was assessed in separate stainings and shown in Fig. 4a. PoFcγRIIa (stained by HuCAL32) was found to be expressed on platelets and a sub-population of eosinophils. The poFcγRIIa/b cross-reactive antibody (HuCAL91) additionally stained a large proportion of monocytes that are thus thought to express poFcγRIIb. Monocytes, neutrophils, and eosinophils all express poFcγRIIIa. Furthermore, small lymphocyte subsets, such as B cells and NK cells are known to express poFcγRIIb and poFcγRIIIa, respectively and monocytes are known to express poFcγRIa (16,27). The poFcγRIa expression beyond lymphocytes and monocytes is largely unknown and can thus not be excluded on platelets, neutrophils, and eosinophils. Histograms in Fig. 4b show the binding of 0.1 μg/ml free huIgG1 and the same amount of huIgG1 complexed using different ratios of VEGF165 to the different minipig blood cell subsets. The antibody (bevacizumab, Bev) to target (VEGF) ratio of 1:2.5 yielded the largest IC without free huIgG1 whereas IC generated in the ratio of 1:0.5 and 1:0.1 were smaller and contained more free huIgG1 (Fig. S5). Here, we observed that large IC showed enhanced binding to all platelets and most monocytes *versus* smaller IC and free huIgG1. Furthermore, large IC resulted in the strongest shift of neutrophils and eosinophils, even though the MFI was lower than in platelets and monocytes. A small subpopulation of lymphocytes also bound large IC better than small IC and free huIgG1 (Fig. 4b). As in the histograms, it is apparent from the titration of all IC preparations in the blood of three Göttingen minipigs that free- and immune-complexed huIgG1 exhibit the strongest binding to platelets, followed by monocytes, eosinophils, neutrophils and lastly lymphocytes (Fig. 4c). The titration shows that in particular the largest IC strongly bind to poFcγR-expressing cell types at the lowest concentrations translating to the highest affinity. Vice versa, preparations with limited VEGF165 or without VEGF165 (huIgG1 alone) require higher concentrations to bind to poFcγR-expressing cell types, translating to lower affinities. VEGF165 did not bind to minipig blood cells at the concentration used to generate the largest IC (ratio 1:2.5) containing 10 μg/ml huIgG1. The strongest differences between free-and immune-complexed huIgG1 were observed in platelets and monocytes. Neutrophils and eosinophils also bound IC stronger than free huIgG1, however the maximum percentage of positive cells in these cell types were lower and the individual differences were more pronounced leading to a higher standard deviation (Fig. 4c).

## Discussion

The use of the Göttingen minipig in preclinical studies with therapeutic antibodies is limited by the lack of knowledge on the expected pharmacology for the translatability of corresponding findings to the human. The pharmacology of antibodies with active Fc parts often depends on effector mechanisms mediated by interaction with FcγRs. The aim of this study was to assess the binding properties of huIgG1 therapeutic antibodies to poFcγRs which is a prerequisite for the consideration of the minipig for preclinical safety and efficacy studies with therapeutic antibodies.

The present study demonstrates that poFcγRs bind human therapeutic antibodies of the IgG1 isotype. The binding properties of the poFcγRIa, poFcγRIIa, and poFcγRIIb closely resemble those of the human orthologues albeit some differences were identified. Importantly, poFcγRIIIa was shown not to bind huIgG1 antibodies. Similar to huFcγRs, all poFcγRs except poFcγRIIIa were shown to bind IC composed of huIgG1 with a higher affinity than free huIgG1. Especially, monocytes, eosinophils, platelets and a subset of lymphocytes of minipig blood showed enhanced binding to human IC.

The poFcγRIa was cloned by Zhang, Qiao (15) and shown to bind poIgG. Here we also demonstrate that poFcγRIa, similar to its human orthologue, strongly binds huIgG1 (28). The high affinity interaction of huFcγRIa is supposed to be mediated by a hydrophobic pocket for Leu235 within the Fc part of huIgG (29). The same pocket was also identified in poFcγRIa supporting its high affinity for huIgG1 (Fig. S2). Nevertheless, we found differences between the two species in residues forming H bonds (Lys128, Ala143 in Fig. S2). This could explain the weaker binding of huIgG1 to poFcγRIa compared to huFcγRIa. The observed difference concerning the heterogeneity of interactions probably results from avidity effects caused by FcγR aggregation. PoFcγRIa is, like its human orthologue, expressed on monocytes in peripheral blood of minipigs (16). A fraction of minipig monocytes binds huIgG1 IC already at low concentrations, possibly mediated by poFcγRIa, although poFcγRIIb and poFcγRIIIa cannot be excluded since these FcγRs are also expressed on monocytes. The enhanced binding of complexed *versus* free huIgG1 to poFcγRIa was confirmed by SPR. Notably, we did not observe a strong staining with free huIgG1 as it could be expected for binding to poFcγRIa. A gradual dissociation of free IgG1 from huFcγRIa is believed to allow capturing small IC or sparsely opsonized large complexes (30). Our results suggest a similar role of poFcγRIa by the observation of the strong IC binding (Fig. 3c and d) and weak staining of poFcγRIa expressing monocytes with low concentrations of free huIgG (Fig. 4b).

FcγRIIa is known as a low affinity receptor signaling through an integrated intracellular immunoreceptor tyrosine-based activation motif (ITAM) in the human. However, orthologues to FcγRIIa in the mouse, cattle and pig, for example, are lacking this integrated ITAM and require FcR γ-chain interactions for signaling (16,31). In terms of binding, we found that FcγRIIa of both species bind huIgG1 (Fig. 1). Conserved tryptophan residues Trp104 and Trp127 forming the “Trp sandwich” of FcγRs that interacts with Pro329 of IgG Fc parts could enable such cross-species interactions (Fig. S2) (32). Interestingly, trastuzumab bound to poFcγRIIa with an increased stability compared to huFcγRIIa (Fig. 1). HEK293F cells expressing porcine and human FcγRIIa showed similarities in binding properties to bevacizumab as observed by the concentration-dependent increase of binding (Fig. 2b). The differences in background and the intensity of the positive population possibly originate from the lower expression of poFcγRIIa compared to huFcγRIIa on HEK293F cells (Fig. 2). A high avidity binding of IC to poFcγRIIa was observed by SPR as described for human low affinity receptors (Fig. 3c and d) (33). This was also reflected in the strong binding of IC to minipig platelets expressing highest levels of FcγRIIa (Fig. 4a). Yet, platelets were also the strongest binders of free bevacizumab. This could be explained by the increased affinity of poFcγRIIa in relation to huFcγRIIa. The increased affinity could further lead to an enhanced sensitivity of the minipig for FcγRIIa binding and ultimately to an overprediction of FcγRIIa-mediated toxicities in preclinical studies. Lau, Gunnarsen (34) observed platelet aggregation and toxicities in domestic pigs treated with mouse IgG2b anti-porcine CD14 (clone MIL2) possibly due to FcγR activation and complement binding. A recombinant huIgG2/4 anti-porcine CD14 antibody (rMIL2) however, did not induce aggregation probably due to abolished FcγR or complement binding in pigs.

The inhibitory low affinity FcγRIIb is mainly expressed on human B cells, dendritic cells (DC), and tissue macrophages and is an important regulator of immune responses (1). Here, we report enhanced binding of poFcγRIIb to trastuzumab in comparison with huFcγRIIb (Fig. 1). The Interaction Map analysis shows a homogeneous interaction for the porcine and human FcγRIIb. Therefore, we conclude that the three times increased affinity is not assay dependent. This finding is also in concordance with *Macaca nemestrina* FcγRIIb showing enhanced huIgG1 binding (32). FcγRIIb of macaques and cyno have residues His131 at the location of the huFcγRIIa His131Arg polymorphism and Met132 nearby. These residues were shown to account for the increased binding while huFcγRIIb has Arg131 and Ser132 in these positions. In poFcγRIIb, however, we identified residues Tyr and Val at the corresponding positions probably influencing the binding in another way (Fig. S2). Triggering of the inhibitory huFcγRIIb in macrophages and dendritic cells can counteract the effects mediated by activating FcγRs (35). Enhanced binding of huIgG1 to poFcγRIIb could therefore enhance the threshold for cell activation and result in a more tolerogenic milieu in inflamed tissue, thus leading to an overestimated efficiency of immunosuppressive therapeutic antibodies in minipigs. Simultaneously, treatment with therapeutic huIgG1 antibodies could lead to enhanced risk for pneumococcal peritonitis while reducing pathological immune stimulation due to reduced reactivity of macrophages (36,37). Furthermore, FcγRIIb expressed on B cells plays an important role in maintenance of peripheral tolerance (38). Thus, the stronger binding of huIgG1 antibodies to poFcγRIIb on B cells could lead to enhanced tolerance and hence to underestimation of immunogenicity concerns.

From all studied receptors, the most pronounced differences between minipig and human were observed for FcγRIIIa. In humans, FcγRIIIa is a low affinity activating receptor binding huIgG1 IC with high avidity and mediating important functions such as ADCC of monocytes and natural killer (NK) cells. PoFcγRIIIa, in contrast, binds neither free- nor immune-complexed huIgG1, and poIgG1a only with low affinity. This binding pattern was observed with recombinant soluble poFcγRIIIa in SPR assays with trastuzumab and with HEK293F cells and neutrophils expressing poFcγRIIIa in interaction with bevacizumab (Figs. 1, 2 and 4). The nature of the poor binding properties of poFcγRIIIa is unknown. However, we cannot exclude binding to other porcine or human IgG subclasses. Similarly, it is known that huIgG isotypes bind differently to mouse FcγRs than mouse IgG isotypes (39). The strong surface expression of FcγRIIIa on porcine monocytes, eosinophils, neutrophils and NK cells suggests important roles for effector functions involving these cell types. Possibly, poFcγRIIIa mediates such functions in with poIgG1a IC or in association with other poIgG isotypes. Indeed, 11 Ig heavy constant *gamma* (IGHG) genes coding for six different IgG subclasses exist in pigs whose specific functions are still unknown (40).

Interestingly, an influenza virus study in landrace cross pigs by Morgan, Holzer (41) reported a lack of efficacy of a hemagglutinin-specific huIgG1 antibody that was expected to reduce the viral load via FcγR-interaction. The mechanistic investigation by flow cytometry revealed no significant binding of free- and immune-complexed huIgG1 to porcine peripheral blood mononuclear cells including lymphocytes and monocytes, even though a slight elevation of positive cells was observed with IC. However, the results from the present study show that large IC, but not free huIgG1 below 10 μg/ml bind to monocytes and weakly to a lymphocyte subset (Fig. 4). These results are difficult to compare to our study due to the unknown huIgG1 concentration, unreported gating, and uncharacterized IC in the publication. Importantly, Morgan, Holzer (41) have shown that the therapeutic huIgG1 antibody does not elicit ADCC by porcine PBMCs and thus concluded a lacking interaction between huIgG1 and all poFcγRs. The present study confirms the lacking interaction between huIgG1 and poFcγRIIIa, that is an important mediator of ADCC in monocytes and NK cells. Nevertheless, we found that huIgG1 antibodies bind to all other poFcγRs. Even though no reduction of the viral load was observed due to lacking ADCC, the said study reported reduced gross pathology (decreased surface of lung lesion) with the hemagglutinin antibody and the huIgG1 control. As proposed before, this finding could be explained by the strong binding huIgG1 to poFcγRIIb and the expression of this receptor on porcine monocytes. The inhibitory function of poFcγRIIb could thus lead to a monocyte-mediated anti-inflammatory effect in interaction with huIgG1 complexes and therefore to reduced tissue damage. On the other hand, the inhibition could be another reason for the unaffected viral load in addition to the lack of NK cell-mediated ADCC.

## Conclusion

In this study, we identified similarities and differences between porcine and human FcγRs regarding binding to huIgG. Taken together, we inferred proper FcγR-mediated effector functions upon treatment of minipigs with human therapeutic antibodies. Due to the similar binding properties of FcγRIa, FcγRIIa, and FcγRIIb we suggest the minipig as a valuable species for assessment of IC-mediated toxicities such as bevacizumab induced platelet activation. The limitations of the minipig relate to the failure of poFcγRIIIa to bind huIgG1 antibodies to mediate effects such as ADCC as demonstrated by the influenza study in pigs with a huIgG1 antibody discussed before (41). Because minipig NK cells express poFcγRIIIa as the only FcγR, we conclude that this cell type cannot mediate ADCC and other effector functions via huIgG1. However, monocyte-mediated effector functions cannot be excluded with huIgG1 because this cell type expresses other FcγRs in addition to poFcγRIIIa. Nevertheless, a reduced or lacking efficacy of huIgG1 antibodies is expected in the minipig. Furthermore, as in most animal species for preclinical studies, also FcγRIIIb-mediated effects of neutrophils, such as acute infusion reactions, cannot be predicted in the minipig due to the unique expression of FcγRIIIb in the human (42). However, the minipig is well suited for pharmacodynamic (PD) studies with therapeutic antibodies as comparable binding strengths of huIgGs were observed to the neonatal Fc receptor (FcRn) between minipigs and humans (43). Nevertheless, it has to be mentioned that the selection of the Göttingen minipig for preclinical studies is dependent on the pharmacological activity of the therapeutic antibody and thus cross-reactivity with the porcine target is required. Furthermore, *in vitro* functional studies and activity assays should be performed to assess the pharmacology of a particular therapeutic antibody prior to the selection of the minipig for preclinical studies.

Here we have described for the first time the cloning and expression of poFcγRIIa, as well as the binding pattern of human therapeutic antibodies to all poFcγRs. The Interaction Map analysis used in this study is a tool to understand complex binding mechanisms *in vitro* and highlights the complexity of FcγR-IgG interactions. Furthermore, it relativizes statements about FcγR affinities in interaction with IgG. Additionally, many novel special formats of therapeutic antibodies are often Fc engineered for altered FcγR binding influencing their mode of action. The binding properties of these novel antibody formats to minipig FcγRs can thus not easily be predicted from our data and will have to be established in a case by case evaluation. The experimental systems described here provide a suitable basis of tools for such evaluation.

### ACKNOWLEDGMENTS AND DISCLOSURES

The authors J.E, T.S, C.S, S.S, D.S, and A.I are employees of F. Hoffmann-La Roche, Ltd. No further funding was received from funding agencies in the public, commercial, or not-for-profit sectors. The authors acknowledge Petra Rüger, Hubert Hertenberger, Daniel Mona, and Dominique Burger for assistance in protein purification and analysis, Martin Nussbaumer for help in protein expression, and Guy Georges for molecular modeling.

## Electronic supplementary material

Fig. S1 Deglycosylation analysis of soluble poFcγRs. Purified soluble poFcγRs were treated with (+) or without (−) PNGase F and analyzed by SDS-PAGE. The molecular weight marker (M) is labeled with the corresponding sizes in kDa on the left of each band. Arrows highlight the reduction of the estimated size after deglycosylation. (PPTX 6048 kb)

Fig. S2 Alignment of the Ig-like C2-type 2 domain (extracellular domain 2) of human, cyno, porcine, cattle and mouse FcγRs. Conserved Trp residues (Trp104 and Trp127 in huFcγRIa) found to be important for interaction with Pro293 of huIgG antibodies are indicated by arrowheads (▼). Residues marked with a diamond shape (◊) form hydrogen bonds between huFcγRIa and huIgG1 and the black circle (●) indicates the hydrophobic pocket huFcγRIa for Leu235 of IgG Fc (27). The asterisk (*) marks the position of the R131H polymorphism in huFcγRIIa influencing its affinity. The poFcγRIIb1 isoform differs from the displayed poFcγRIIb in the two amino acid residues highlighted in yellow (His153Asn and Asn168Asp). Sequences used for this MUSCLE alignment are: Human FcγRIa (Uniprot: P12314), FcγRIIa (Uniprot: P12318), FcγRIIb (Uniprot: P31994), FcγRIIIa (Uniprot: P08637); cyno FcγRI (Uniprot; Q8SPW5), FcγRIIa (Uniprot; Q8SPW4), FcγRIIb (Uniprot; Q8SPW3), FcγRIII (Uniprot; Q8SPW2); porcine FcγRIa (Uniprot; Q461Q0), FcγRIIa (Transcript XM_021089520), FcγRIIb (Uniprot; B9VVN4), FcγRIIIa (Uniprot; Q28942); cattle FcγRIa (Uniprot: Q9MZT0), FcγRIIa (Uniprot: A8DC37), FcγRIIb (Uniprot: Q28110), FcγRIII (Uniprot: P79107); mouse FcγRIa (Uniprot: P26151), FcγRIII (Uniprot: P08508), FcγRII (Uniprot: P08101), FcγRIV (Uniprot: Q3TC44). (PPTX 1062 kb)

Fig. S3 SPR binding analysis comparing porcine FcγRIIb and its sub-isoform FcγRIIb1. This figure is analogous to Fig. 1b and c. The real-time sensorgrams from SPR analysis in the upper row show interaction of HER2-specific huIgG1 (trastuzumab, red), poIgG1a-HER2 (green), and poIgG3-HER2 (blue) with the respective FcγR named above. A titration with 600, 200, and 66.7 nM of soluble FcγR is shown binding the antigen-bound IgG on the chip surface. Interaction Map analysis resulting from trastuzumab binding to all concentrations of porcine FcγRs is shown in the lower row. The binding is separated in its parallel interactions with unique kinetics, as displayed by spots on a graph with k_d_ on the x-axis and k_a_ on the y-axis. The heatmap is a measure of the contribution (red = high, blue = low) of each interaction to the total binding. (PPTX 1373 kb)

Fig. S4 Gating strategy for flow cytometry analysis of minipig blood. Whole blood from Göttingen minipigs was stained with the indicated fluorochrome-labeled antibodies. From singe and live cells, gates P1-P5 were selected using forward (FSC) and side scatter (SSC) and cell types were identified using the following antibody clones: CD45 (K252.1E4), CD61 (JM2E5), CD3e (BB23-8E6-8C8), CD21 (BB6-11C9.6), CD335 (VIV-KM1), CD8a (76-2-11), CD172a (74-22-15A), CD14 (MIL2), and CD52 (11/305/44). Numbers indicate the percentage of cells within the respective population (P1-P5). (PPTX 1084 kb)

Fig. S5 Size distribution of the immune complex preparations. IC preparations generated by different molar ratios of antibody (bevacizumab, Bev, huIgG1) to target (VEGF165 dimer, VEGF) were assessed by SEC-MALS. The IC generated by the bevacizumab to VEGF165 ratio of 1:2.5 (red line) does not contain free huIgG1 (as apparent at a molar mass of 1.4 × 10^5^ g/mol), whereas the ratio of 1:0.5 (magenta line) contains approx. 40% of free huIgG1, and the ratio of 1:0.1 (purple line) contains approx. 85% of free huIgG1. Bevacizumab alone (ratio 1:0, blue line) and VEGF165 dimers alone (ratio 0:1, green line) did not form any large complexes. (PPTX 3272 kb)

## Abbreviations

ADCC: Antibody-dependent cellular cytotoxicity
ADCP: Antibody-dependent cellular phagocytosis
FcγR: Fc *gamma* receptor protein
*FCGR*: Fc *gamma* receptor gene
FcR-γ chain: Fc receptor common *gamma* chain
HuCAL: Human combinatorial antibody library
IC: Immune complex
ITAM: Immunoreceptor tyrosine-based activation motif
NHP: Non-human primate
SPR: Surface plasmon resonance
