# Supplementary figures and images for: The Binding of Human IgG to Minipig FcγRs – Implications for Preclinical Assessment of Therapeutic Antibodies
Source: Pharm Res. 2019 Feb 5;36(3):47. doi: 10.1007/s11095-019-2574-y (PMC6373530; doi:10.1007/s11095-019-2574-y)

## Slide 1
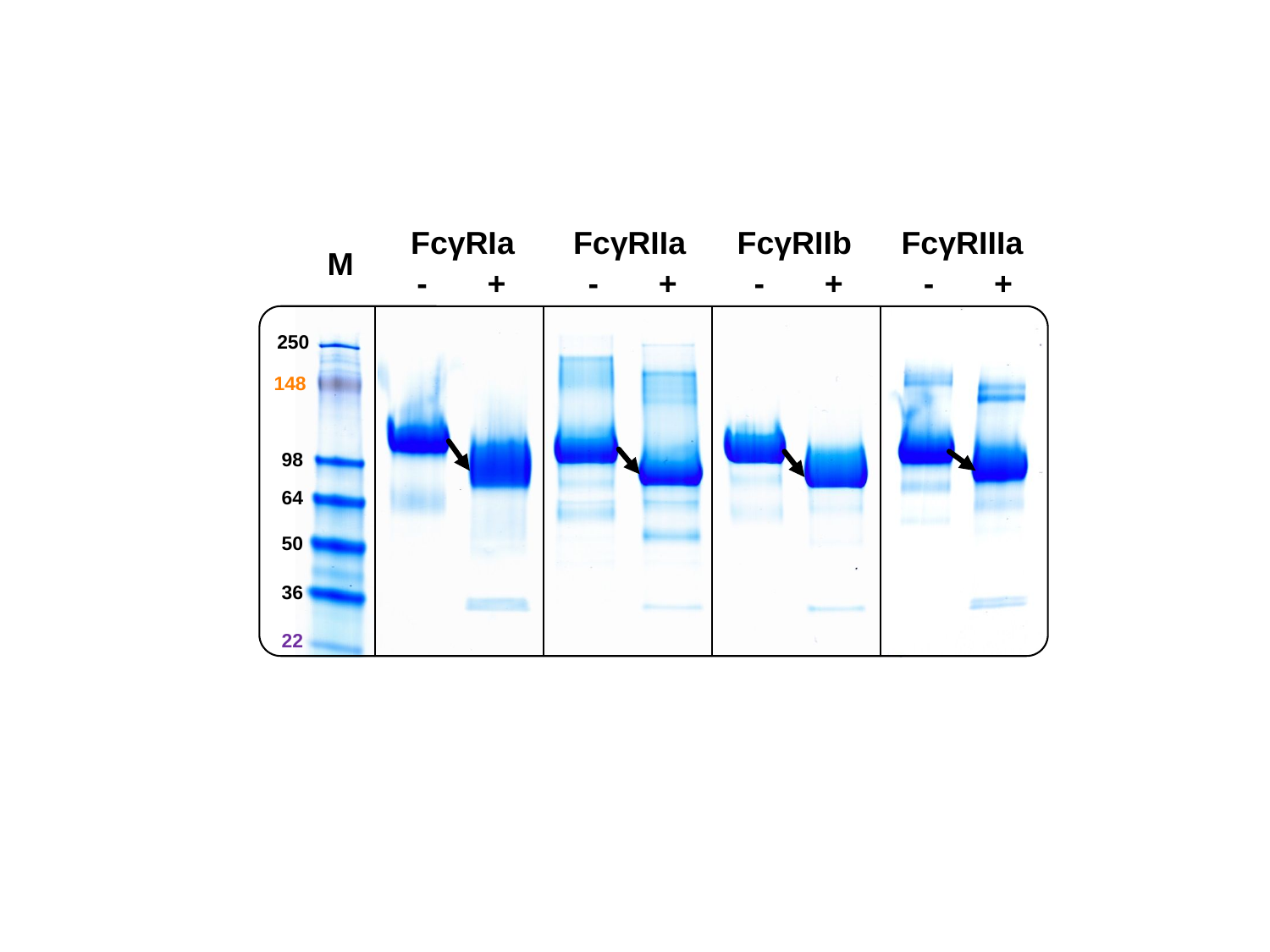

FcγRIa
FcγRIIa
FcγRIIb
FcγRIIIa
M
-
+
-
+
-
+
-
+
250
148
98
64
50
36
22

Supplement: Supplementary file 1 — Deglycosylation analysis of soluble poFcγRs. Purified soluble poFcγRs were treated with (+) or without (−) PNGase F and analyzed by SDS-PAGE. The molecular weight marker (M) is labeled with the corresponding sizes in kDa on the left of each band. Arrows highlight the reduction of the estimated size after deglycosylation. (PPTX 6048 kb) [file 11095_2019_2574_MOESM1_ESM.pptx]

## Slide 1
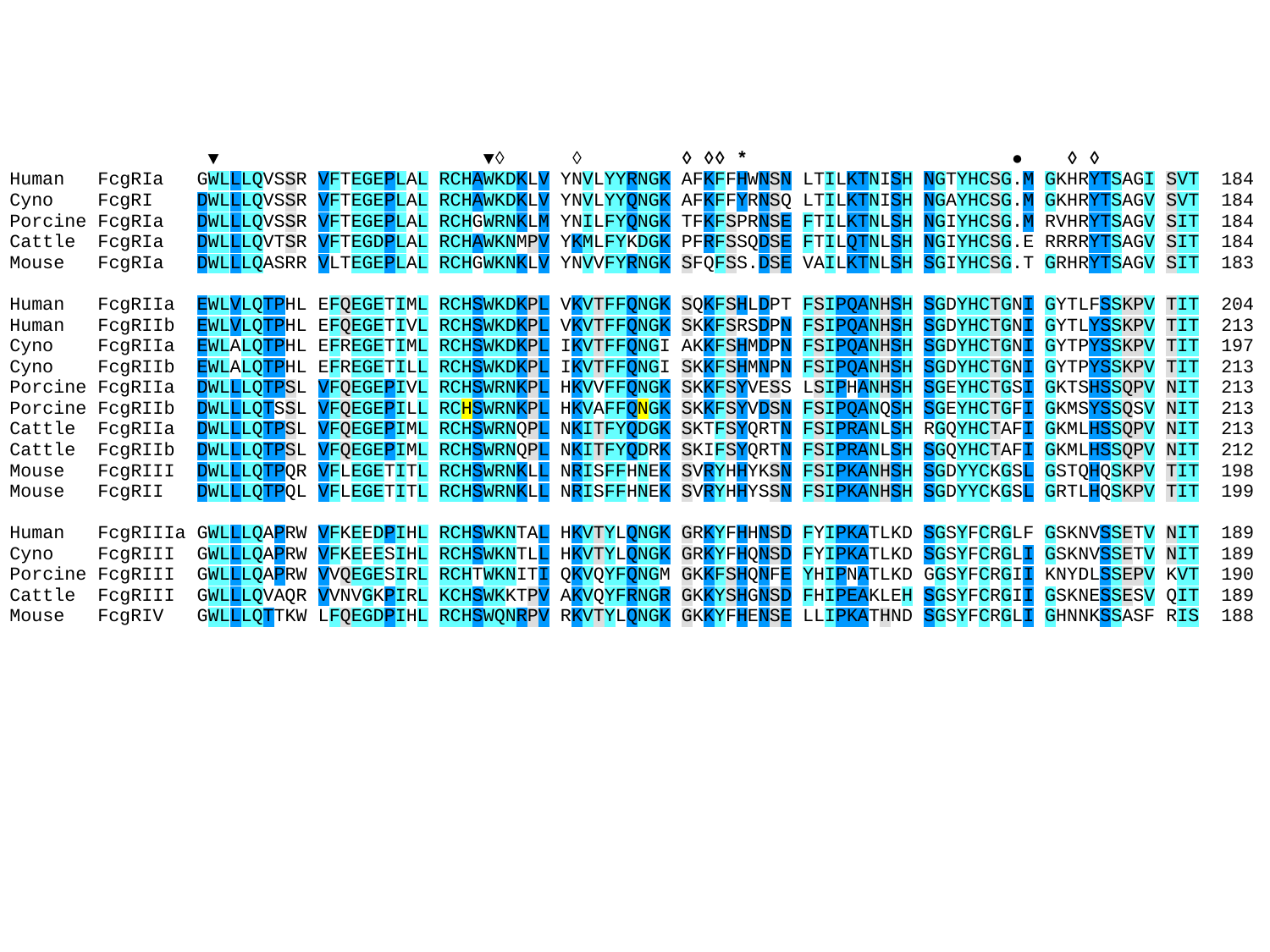

Supplement: Supplementary file 2 — Alignment of the Ig-like C2-type 2 domain (extracellular domain 2) of human, cyno, porcine, cattle and mouse FcγRs. Conserved Trp residues (Trp104 and Trp127 in huFcγRIa) found to be important for interaction with Pro293 of huIgG antibodies are indicated by arrowheads (▼). Residues marked with a diamond shape (◊) form hydrogen bonds between huFcγRIa and huIgG1 and the black circle (●) indicates the hydrophobic pocket huFcγRIa for Leu235 of IgG Fc (27). The asterisk (*) marks the position of the R131H polymorphism in huFcγRIIa influencing its affinity. The poFcγRIIb1 isoform differs from the displayed poFcγRIIb in the two amino acid residues highlighted in yellow (His153Asn and Asn168Asp). Sequences used for this MUSCLE alignment are: Human FcγRIa (Uniprot: P12314), FcγRIIa (Uniprot: P12318), FcγRIIb (Uniprot: P31994), FcγRIIIa (Uniprot: P08637); cyno FcγRI (Uniprot; Q8SPW5), FcγRIIa (Uniprot; Q8SPW4), FcγRIIb (Uniprot; Q8SPW3), FcγRIII (Uniprot; Q8SPW2); porcine FcγRIa (Uniprot; Q461Q0), FcγRIIa (Transcript XM_021089520), FcγRIIb (Uniprot; B9VVN4), FcγRIIIa (Uniprot; Q28942); cattle FcγRIa (Uniprot: Q9MZT0), FcγRIIa (Uniprot: A8DC37), FcγRIIb (Uniprot: Q28110), FcγRIII (Uniprot: P79107); mouse FcγRIa (Uniprot: P26151), FcγRIII (Uniprot: P08508), FcγRII (Uniprot: P08101), FcγRIV (Uniprot: Q3TC44). (PPTX 1062 kb) [file 11095_2019_2574_MOESM2_ESM.pptx]

## Slide 1
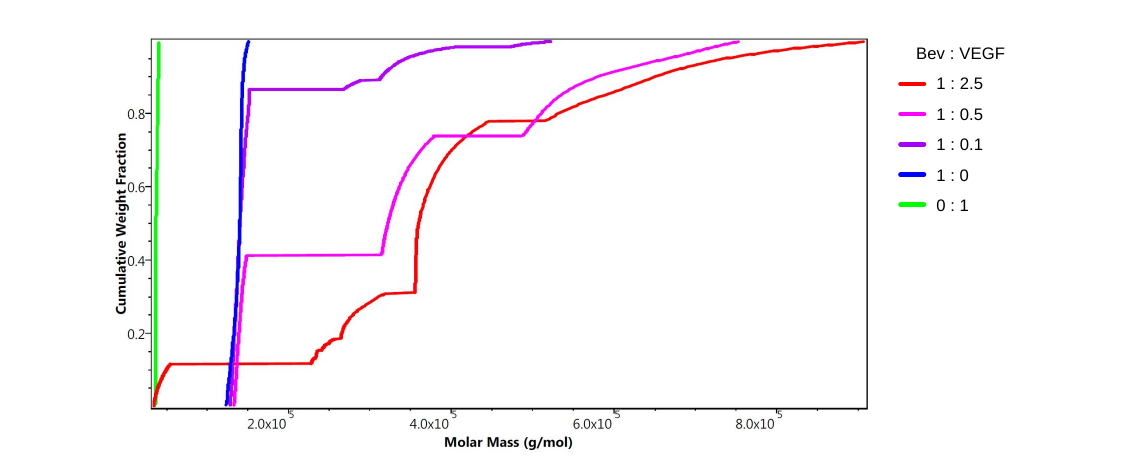

Bev : VEGF
1 : 2.5
1 : 0.5
1 : 0.1
1 : 0
0 : 1

Supplement: Supplementary file 5 — Size distribution of the immune complex preparations. IC preparations generated by different molar ratios of antibody (bevacizumab, Bev, huIgG1) to target (VEGF165 dimer, VEGF) were assessed by SEC-MALS. The IC generated by the bevacizumab to VEGF165 ratio of 1:2.5 (red line) does not contain free huIgG1 (as apparent at a molar mass of 1.4 × 105 g/mol), whereas the ratio of 1:0.5 (magenta line) contains approx. 40% of free huIgG1, and the ratio of 1:0.1 (purple line) contains approx. 85% of free huIgG1. Bevacizumab alone (ratio 1:0, blue line) and VEGF165 dimers alone (ratio 0:1, green line) did not form any large complexes. (PPTX 3272 kb) [file 11095_2019_2574_MOESM5_ESM.pptx]
