# Supplementary material for: The Binding of Human IgG to Minipig FcγRs – Implications for Preclinical Assessment of Therapeutic Antibodies
Source: Pharm Res. 2019 Feb 5;36(3):47. doi: 10.1007/s11095-019-2574-y (PMC6373530; doi:10.1007/s11095-019-2574-y)
Supplement: Supplementary file 3 — SPR binding analysis comparing porcine FcγRIIb and its sub-isoform FcγRIIb1. This figure is analogous to Fig. 1b and c. The real-time sensorgrams from SPR analysis in the upper row show interaction of HER2-specific huIgG1 (trastuzumab, red), poIgG1a-HER2 (green), and poIgG3-HER2 (blue) with the respective FcγR named above. A titration with 600, 200, and 66.7 nM of soluble FcγR is shown binding the antigen-bound IgG on the chip surface. Interaction Map analysis resulting from trastuzumab binding to all concentrations of porcine FcγRs is shown in the lower row. The binding is separated in its parallel interactions with unique kinetics, as displayed by spots on a graph with kd on the x-axis and ka on the y-axis. The heatmap is a measure of the contribution (red = high, blue = low) of each interaction to the total binding. (PPTX 1373 kb) [file 11095_2019_2574_MOESM3_ESM.pptx]

## Slide 1
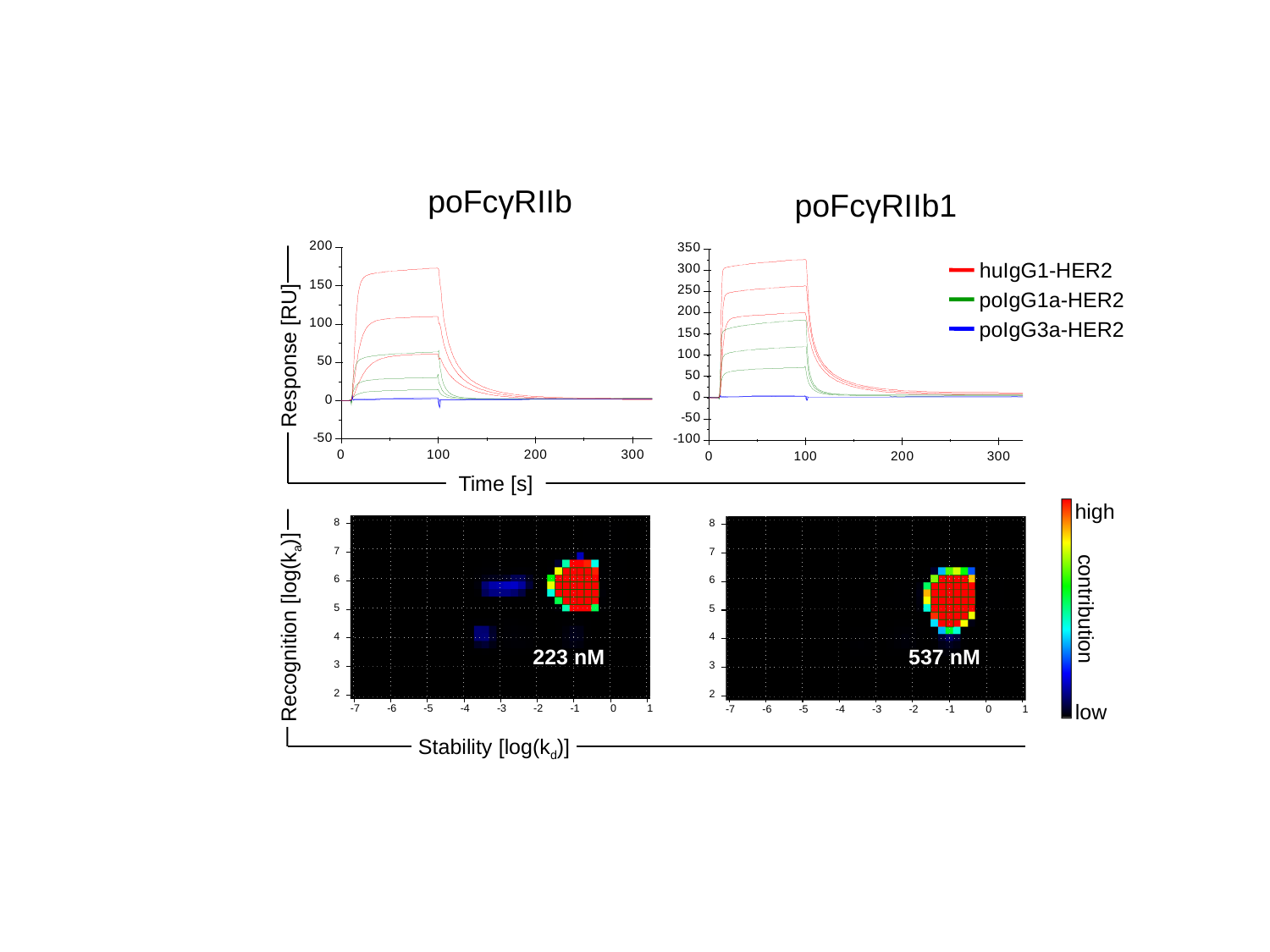

poFcγRIIb
poFcγRIIb1
huIgG1-HER2
poIgG1a-HER2
poIgG3a-HER2
Response [RU]
Time [s]
high
8
7
6
5
4
3
2
-7
-6
-5
-4
-3
-2
-1
0
1
8
7
6
5
4
3
2
-7
-6
-5
-4
-3
-2
-1
0
1
contribution
Recognition [log(ka)]
223 nM
537 nM
low
Stability [log(kd)]
