# Supplementary material for: The Binding of Human IgG to Minipig FcγRs – Implications for Preclinical Assessment of Therapeutic Antibodies
Source: Pharm Res. 2019 Feb 5;36(3):47. doi: 10.1007/s11095-019-2574-y (PMC6373530; doi:10.1007/s11095-019-2574-y)
Supplement: Supplementary file 4 — Gating strategy for flow cytometry analysis of minipig blood. Whole blood from Göttingen minipigs was stained with the indicated fluorochrome-labeled antibodies. From singe and live cells, gates P1-P5 were selected using forward (FSC) and side scatter (SSC) and cell types were identified using the following antibody clones: CD45 (K252.1E4), CD61 (JM2E5), CD3e (BB23-8E6-8C8), CD21 (BB6-11C9.6), CD335 (VIV-KM1), CD8a (76-2-11), CD172a (74-22-15A), CD14 (MIL2), and CD52 (11/305/44). Numbers indicate the percentage of cells within the respective population (P1-P5). (PPTX 1084 kb) [file 11095_2019_2574_MOESM4_ESM.pptx]

## Slide 1
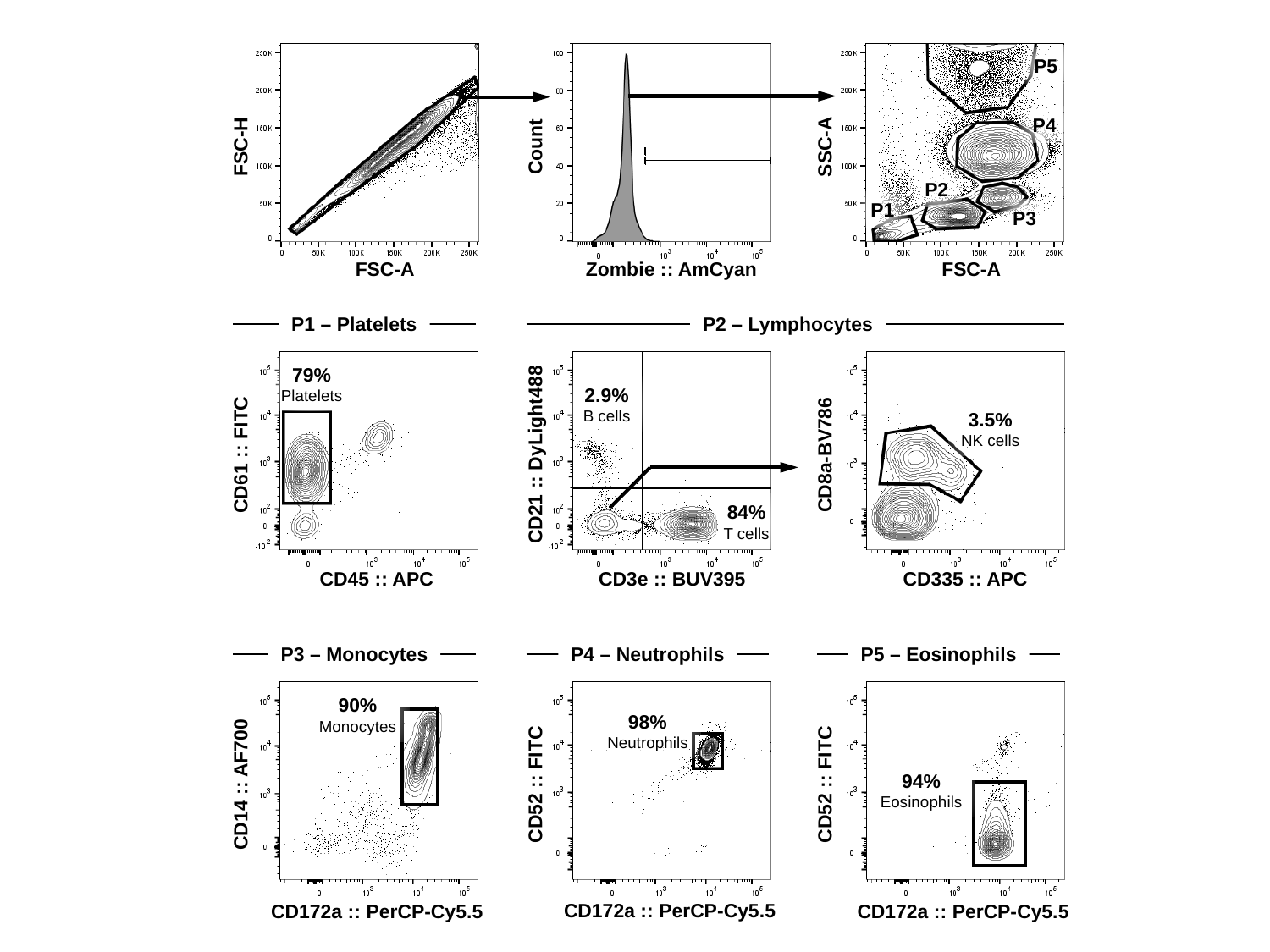

P5
P4
FSC-H
SSC-A
Count
P2
P1
P3
FSC-A
Zombie :: AmCyan
FSC-A
P1 – Platelets
P2 – Lymphocytes
79%
Platelets
2.9%
B cells
3.5%
NK cells
CD61 :: FITC
CD8a-BV786
CD21 :: DyLight488
84%
T cells
CD45 :: APC
CD3e :: BUV395
CD335 :: APC
P3 – Monocytes
P4 – Neutrophils
P5 – Eosinophils
90%
Monocytes
98%
Neutrophils
94%
Eosinophils
CD14 :: AF700
CD52 :: FITC
CD52 :: FITC
CD172a :: PerCP-Cy5.5
CD172a :: PerCP-Cy5.5
CD172a :: PerCP-Cy5.5
